# Multiple Year Influences of the Aflatoxin Biocontrol Product AF-X1 on the *A. flavus* Communities Associated with Maize Production in Italy

**DOI:** 10.3390/toxins15030184

**Published:** 2023-02-28

**Authors:** Mohamed Ali Ouadhene, Alejandro Ortega-Beltran, Martina Sanna, Peter J. Cotty, Paola Battilani

**Affiliations:** 1 Department of Sustainable Crop Production, Università Cattolica del Sacro Cuore, 29122 Piacenza, Italy; 2 International Institute of Tropical Agriculture (IITA), Ibadan 200001, Nigeria; 3 AGROINNOVA—Centre of Competence for the Innovation in the Agro-Environmental Sector, University of Torino, 10095 Grugliasco, Italy; 4 College of Food Science and Engineering, Ocean University of China, Qingdao 266003, China

**Keywords:** biocontrol, vegetative compatibility group, carry-over, non-aflatoxigenic, aflatoxins

## Abstract

AF-X1 is a commercial aflatoxin biocontrol product containing the non-aflatoxigenic (AF-) strain of *Aspergillus flavus* MUCL54911 (VCG IT006), endemic to Italy, as an active ingredient. The present study aimed to evaluate the long-term persistence of VCG IT006 in the treated fields, and the multi-year influence of the biocontrol application on the *A. flavus* population. Soil samples were collected in 2020 and 2021 from 28 fields located in four provinces in north Italy. A vegetative compatibility analysis was conducted to monitor the occurrence of VCG IT006 on the total of the 399 isolates of *A. flavus* that were collected. IT006 was present in all the fields, mainly in the fields treated for 1 yr or 2 consecutive yrs (58% and 63%, respectively). The densities of the toxigenic isolates, detected using the *aflR* gene, were 45% vs. 22% in the untreated and treated fields, respectively. After displacement via the AF- deployment, a variability from 7% to 32% was noticed in the toxigenic isolates. The current findings support the long-term durability of the biocontrol application benefits without deleterious effects on each fungal population. Nevertheless, based on the current results, as well as on previous studies, the yearly applications of AF-X1 to Italian commercial maize fields should continue.

## 1. Introduction

*Aspergillus flavus* Link is one of the most important filamentous fungi worldwide because it can produce aflatoxins in various crops of economic importance. This species is widely distributed in temperate, tropical, and subtropical zones [1], including various regions in Europe [2,3], and thrives in many agro-ecosystems and diverse natural habitats. The competitive advantages of *A. flavus* increase under several abiotic stresses, including its high temperature and salinity [4,5]. However, it is as a causal agent of aflatoxin contamination that *A. flavus* is most frequently distinguished. The International Agency for Research on Cancer [6] classifies aflatoxin B1 (AFB1) as a Group 1 compound because it is known to be carcinogenic to humans [7]. As a result, the maximum levels (MLs) for aflatoxins in food and feed have been established in most countries, to prevent the commercialization and consumption of unsafe commodities [8,9,10].

The life cycle of *A. flavus* is divided into an opportunistic phase, during which plants, animals, and humans are infected, and a saprophytic phase, where detritus is produced from excrement and through the decay of plant and insect parts and other organic matter [1]. Mycelia, conidia, and sclerotia are produced during both phases, providing for reproduction and survival in the absence of the nutrients and/or environments conducive to growth [11,12,13,14]. Many *A. flavus* genotypes produce AFB1 and AFB2, but other genotypes lack the abilities to produce aflatoxins [15]. Based on the morphology of sclerotia, two morphotypes of *A. flavus* have been characterized: S morphotype, known for the production of copious small sclerotia (<400 µm in diameter), and L morphotype, characterized by sclerotia that are fewer in numbers but larger (>400 µm) [16]. Almost all S morphotype strains produce aflatoxin at high concentrations, while the aflatoxin-producing potentials of L morphotype strains vary widely, ranging from highly toxigenic to non-aflatoxigenic (AF−). Genotypes that produce no aflatoxins are termed as non-aflatoxigenic (AF−). The presence of AF- strains has been reported in most studies that evaluate *A. flavus* diversity [16,17,18,19,20]. 

There are many *A. flavus* genetic groups, called Vegetative Compatibility Groups (VCGs), which are delimited by a self/non-self-recognition system [17,18]. VCGs that are composed entirely of AF- members reflect the stable retention of this phenotype during clonal evolution [19]. A lack of aflatoxins production does not affect the ability of *A. flavus* to infect and decay crops. In fact, the aggressiveness of some AF- strains lets them be tested as biocontrol agents with the potential to competitively exclude aflatoxin producers [16]. In 1989, the AF- strain AF36 was applied for the first time to a field in Yuma, Arizona, after being tested at laboratory scale, and it significantly reduced the aflatoxin contamination in cottonseed [16]. 

The displacement of aflatoxin producers is one possible mechanism by which the applied AF- strains reduce aflatoxin contamination [1,20]. From the initial commercial field evaluations of the AF36 strain in 1996, it became evident that AF- biocontrol products shift the *A. flavus* population structure in treated fields, and these changes to the population structure may be retained, in part, over multiple years [21,22]. Similar results also occur in small-scale field studies [23] and in multi-year evaluations of the biocontrol product Aflasafe used in commercial maize fields in Kaduna State, Nigeria [24].

There are currently over 50 AF- strains of *A. flavus* that are registered for use as active ingredients in the aflatoxin biocontrol products used in various countries [25,26]. However, AF-X1 is the only product currently available in the EU. AF-X1 has been used in Italy since 2015. Its active ingredient is *A. flavus* MUCL 54911, which is endemic to Italy and belongs to VCG IT006 [27]. All members of this VCG lack the entire aflatoxin biosynthesis gene cluster, as a result of a large insertion/deletion event shared in common with a number of other AF- genotypes of *A. flavus* [19,28]. 

In 2003, there was an aflatoxin outbreak in the maize grown in North Italy [2], which was ingested by dairy livestock. This resulted in large quantities of milk being destroyed, thereby having a significant impact on the regions’ signature cheese production by the consortia of Parmigiano Reggiano and Grana Padano. Since 2003, North Italy has experienced additional aflatoxin contamination events, as have many temperate regions in southeastern Europe [3,29], causing significant problems for both the maize and dairy industries [30]. Furthermore, climate change is predicted to worsen the maize contamination in Europe [31]. The use of different compounds able to bind AFB1 to reduce its bioavailability was purposed, but this approach is not totally effective and implies side effects [32,33]. A biocontrol formulation comprised on an AF- *A. flavus* strain, AF-X1, was successfully developed to address the contamination in commercially grown maize in Italy. The use of AF-X1 has resulted in substantial relief for the maize industry in Italy, with aflatoxin contamination reduced more than 90% compared to untreated maize [28,34,35]. As the only biocontrol formulation currently available in the EU, AF-X1 has been commercialized with a temporary authorization since 2015, while the final registration work is still ongoing.

There are several possible mechanisms through which the AF- strains of *A. flavus* may impact aflatoxin contamination, such as competitive exclusion [21], nutrient sequestration [36], touch inhibition, or chemosensing (extrolites or VOCs) [37,38]. However, the predominant mechanism reported in agricultural fields is the modification of *Aspergillus* populations through the displacement of aflatoxin-producing potential [39,40,41]. The AF- strains displace the toxigenic strains and thereby reduce the aflatoxin content in many of the crops grown commercially in the United States, Nigeria, Kenya, Senegal, The Gambia, Ghana, and Italy [28,40,42,43,44,45].

Aflatoxin concentration is influenced by a high temperature, low humidity, and precipitation. Moreover, crop rotation and the timing of planting and harvest have strong effects on contamination. These factors make it difficult to determine the multi-year efficacy of biocontrol applications based on the aflatoxin concentrations alone [31,46,47]. In some regions, biocontrol strains have been shown to persist beyond a single growing season and expand their range beyond the treated fields [22,23,44,46,48]. However, the extents of such influences are dependent on both the AF- genotypes employed and the agro-ecosystem in which the product is used [49]. The residual influences of biocontrol products can be assessed by the multi-year monitoring of the AF- active ingredients in agricultural soils. This can be done by examining individual genotypes within the resident fungal population and characterizing those individuals with either culture-based (i.e., a vegetative compatibility analysis (VCAs) [20,50] or molecular tools, such as microsatellite analyses or SNP monitoring with pyrosequencing [51,52,53]. Moreover, several studies have previously identified the role of some aflatoxin biosynthesis pathway genes, such as *omt*-A and *aflR*, to develop new approaches to estimate the aflatoxin-producing capacity of *Aspergillus* spp., such as the use of real-time PCR [54,55,56]. In fact, qPCR was previously used to detect AF36 during pistachio production [57]. In addition, Cluster Amplification Pattern (CAP) is a multiplex PCR method used to monitor the stability of the AF- strains of *A. flavus* [58]. 

Currently, there are no studies on either the long-term efficacy of AF-X1 or the influences of the maize-based agro-ecosystem of northern and central Italy on AF-X1 persistence. Therefore, our study sought to assess the long-term effects of the commercial applications of the biocontrol product AF-X1 on the structure of the *A. flavus* communities’ resident in fields that are frequently cropped to maize in north Italy. The residual influences of these applications may provide cumulative benefits over multiple seasons and may, in part, explain the reduced frequencies of aflatoxin contamination in regions where the applications of AF-X1 were previously employed. 

## 2. Results

### 2.1. Cropping Systems of the Surveyed Fields

Most of the fields (70%) included in the current study contained predominantly silt soil (Table 1). In total, six (21%) fields were predominantly clay, and three (11%) were sandy. Several of the sampled fields (25%) were planted with maize repeatedly, without rotation. However, some of the fields were rotated between maize and either wheat, soybean, tomato (rarely), or pea. Conventional tillage was commonly applied, with conservative approaches (no tillage) reported only for three fields in area 2 of Rovigo. In addition, stalk burial was performed in ~50% of the fields, and all four of the fields sampled in area 6 (Table 1).

### 2.2. Soil Fungal Populations

The total fungal community (Table 2) in the soil sampled in 2020 was significantly (*p* < 0.01) influenced by the AF-X1 application schedule; however, no significant influence was observed in the soils sampled in 2021. Furthermore, the interaction between the treatment and location was significant (*p* < 0.01). 

**Figure 1:**
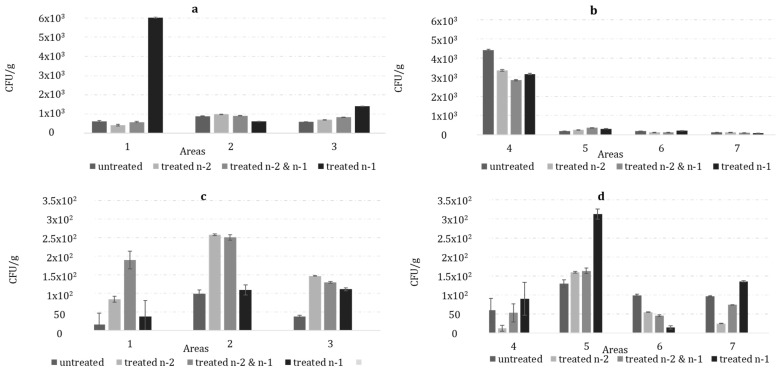
Colony-forming unit (CFU/g + confidence interval) of total fungal community resident in the soil samples collected from 3 areas in 2020 (**a**); 4 areas in 2021 (**b**); of *A. flavus* in the same soil sample collected in 2020 (**c**); and in 2021 (**d**). The 7 sampling areas belong to 4 districts: 1 (Rovigo), 2 (Rovigo), and 3 (Rovigo) in 2020, and 4 (Modena), 5 (Rovigo), 6 (Padova), and 7 (Venezia) in 2021. In each area 4 different treatment regimens were applied: untreated, treated n-2, treated n-2&n-1, and treated n-1; n is the sampling year.

The incidences of the total fungal occurrence (CFU/g) were calculated for each field sampling site (Figure 1), and the distribution of the total fungal population varied among them. The highest fungal occurrence was noted in area 4 with 3443 CFU/g, and the lowest in area 7 with 112 CFU/g (Figure 1b). Additionally, a CFU/g increase of 47% was noted in all the fields treated n-1 compared to the untreated fields. The distribution of the total fungal population varied within the areas. The highest fungal concentrations occurred in a field treated n-1 in area 1 (6027 CFU/g).

The occurrence of *A. flavus* in each field site also varied with treatment (*p* < 0.01), being inconsistent and ranging from 51 to 190 CFU/g (Table 2). Overall, the results from both sampling years indicated that the lowest average recovery of *A. flavus* (51 CFU/g) occurred in an untreated field in 2020 with 51 CFU/g (Figure 1c). The concentrations of *A. flavus* were elevated in both the fields treated in a single year and the fields treated over two years (2020–2021; Table 1). There was a significant interaction between the treatments and locations, but only for the data collected in 2021 (*p* < 0.01).

The only time an untreated field site had a greater abundance of *A. flavus* than the treated neighbor fields was in the Noale municipality (sampling year 2021), whereby the untreated field had the greatest *A. flavus* concentrations (99 CFU/g), and all of the three treated sites were ≤50 CFU/g (Figure 1d).

### 2.3. Frequency of Non-Alfatoxigenic and Toxigenic A. flavus

Overall, 399 *A. flavus* isolates were collected from the soil samples. The frequency of isolates lacking the *aflR* gene was determined using a qPCR method and found to be 287 (72%) (Table 2). As expected, the occurrence of the AF- isolates lacking the *aflR* gene was significantly influenced by AF-X1 treatment (*p* < 0.05), but only when the two sampling years were combined; the fields treated for two years had significantly more AF- isolates compared to the untreated fields.

**Figure 2:**
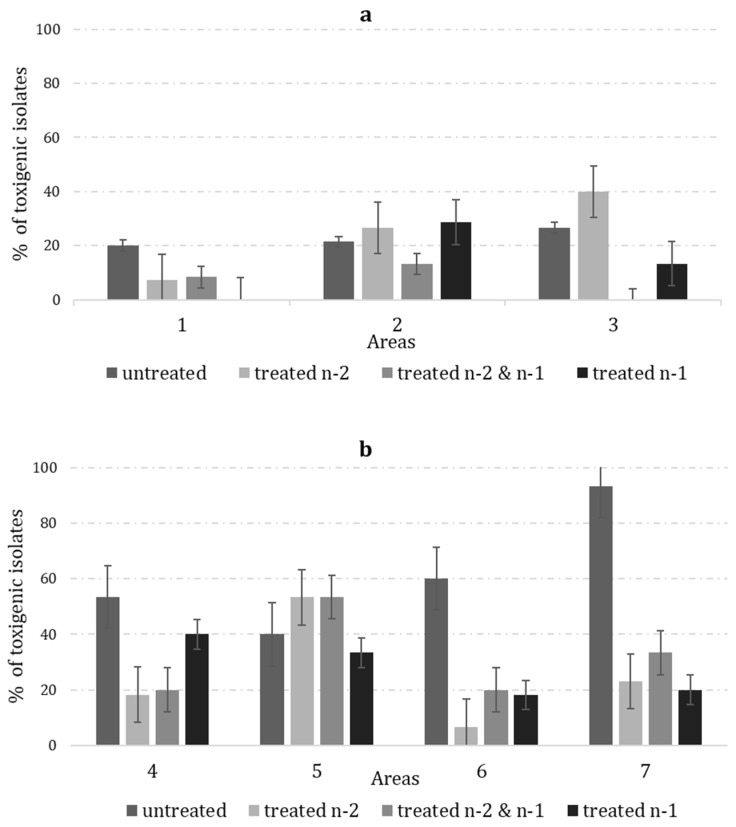
Percentage of toxigenic isolates of *A. flavus* isolates in the soil samples collected from 3 areas in 2020 (**a**); and 4 areas in 2021 (**b**). The 7 sampling areas belong to 4 districts: 1 (Rovigo), 2 (Rovigo), and 3 (Rovigo) in 2020, and 4 (Modena), 5 (Rovigo), 6 (Padova), and 7 (Venezia) in 2021. In each area, 4 different treatment regimens were applied: untreated, treated n-2, treated n-2&n-1, and treated n-1; n is the sampling year.

The highest occurrence of isolates lacking *aflR* was noted in all the treated fields and ranged from 68% to 93% among the examined communities of *A. flavus* (Table 2). However, a lower overall occurrence of toxigenic isolates was sampled in the untreated fields during 2020 compared to the untreated fields sampled in 2021 (Figure 2). 

### 2.4. Frequency of Isolates Belonging to VCG IT006

**Figure 3:**
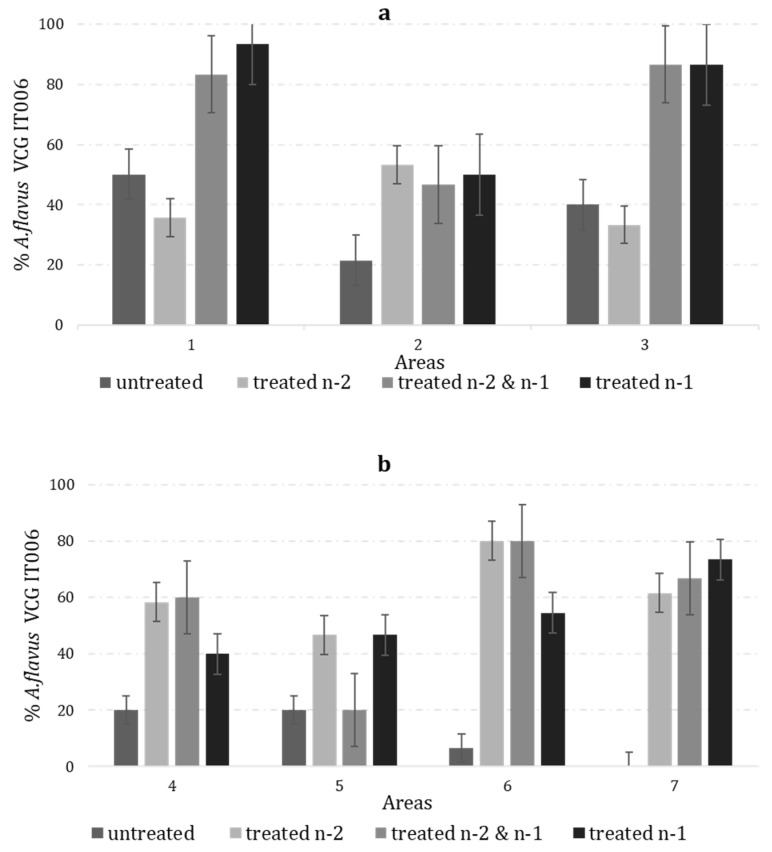
Incidence (%) of *A. flavus* strains belonging to the AF- VCG IT006 on total number of *A. flavus* isolates, the active ingredient of AF-X1 in soil samples collected in 2020 (**a**), and 2021 (**b**); soil samples were collected from 3 areas in 2020 (**a**), and 4 areas in 2021 (**b**), belonging to 4 districts: 1 (Rovigo), 2 (Rovigo), and 3 (Rovigo) in 2020, and 4 (Modena), 5 (Rovigo), 6 (Padova), and 7 (Venezia) in 2021. In each area, 4 different treatment regimens were applied: untreated, treated n-2, treated n-2&n-1, and treated n-1; n is the sampling year.

The 399 *A. flavus* isolates recovered from the soil samples were subjected to classical VCG testing. A total of two hundreds of the isolates shared VCG IT006 with MUCL-54911. A subsequent analysis revealed that they all lacked *aflR*, as expected. The remaining 199 isolates did not belong to IT006, and 84 (42%) of those also did not have *aflR*. The abundance of VCG IT006 isolates was significantly influenced by the AF-X1 treatment regimen (*p* < 0.01), but only in the fields sampled in 2021. VCG IT006 was significantly lower in the untreated fields, both when the incidence was computed for the total *A. flavus* or AF- isolates (Table 2). When the counts for both the sampling years were combined, the significantly lower incidence of VCG IT006 in the untreated fields was confirmed. Similar ranges of IT006 frequency were observed in the fields treated two years prior (20% to 83% IT006) and one year prior to sampling (40% to 93% IT006) (Figure 3). However, the frequencies of IT006 in the untreated field areas 1 and 3 (2020) were more than double the amounts recovered in any other untreated field, as well as in those treated in 2018 (+29% vs. +17%) (Figure 3a).

### 2.5. Impact of Cropping System on the Soil Fungal Population

The cropping conditions that were available and collected from farmers were crop rotation, the soil type, tillage (most of the fields were under conventional tillage), and stalk burial, which in all studied fields was not applied. Crop rotation was the only factor among the cropping system that significantly influenced the fungal population isolated from the soil, with a significantly higher CFU/g and wheat grown before maize compared to soybean. The incidence of the AF- isolates, just as the incidence of IT006, was the highest with maize as the preceding crop (data not shown). 

## 3. Discussion

Farmers, industries, and regulatory authorities have questioned if the applications of the aflatoxins biocontrol product might have long-term benefits [34,35]. The current study provides observations that suggest that the applications of AF-X1 have influences that extend to the next season and the season after, and even to nearby neighboring untreated fields. The soils collected in 2020 and 2021 from the fields located in northern Italian maize production areas, where AF-X1 was previously applied, contained significant frequencies of the VCG, to which MUCL 54911, the active ingredient of AF-X1, belongs. The results (Table 2) indicate that: (I) the use of AF-X1 has a residual effect that improves the structure of the *A. flavus* resident in both the treated fields and in the neighboring untreated fields, so that the AF- active ingredient is more common and the frequency of the aflatoxin producers is reduced; and (II) the application of AF-X1 promotes the creation of these safer *Aspergillus* populations, with no significant effects on the total fungal communities. These results suggest that follow-up studies should be used to determine the frequencies and distributions of the AF-X1 applications required for the levels of cost-effective aflatoxin management required by north Italy’s maize industry, to provide grain that is consistently safe for the region’s vital dairy industry. To assess the residual effects of AF-X1 applications, VCA was undertaken to assess the abundance of its active ingredient, MUCL 54911, in the current study, despite being a labor-intensive, time-consuming technique; this has been judged the most reliable and accurate method available, and the only method which has been successfully applied to identifying MUCL 54911 in field samples [51,59]. A significant occurrence of MUCL 54911 in all the treated areas was reported. Similarly, the application of single AF- *A. flavus* isolates of the aflatoxin biocontrol products, Afla-Guard^®^ and AF36, resulted in persistence overtime. In addition, the most extensive carry-over studies, involving thousands of isolates, were carried out in the U.S. with AF36 [23,60,61]. A similar carry-over was observed on African small holder farms with Aflasafe, a biocontrol product containing four AF- strains as its active ingredients [24]. 

Several studies have shown not only the survival, but also an increased frequency of AF- biocontrol product VCGs beyond the treatment season [44,60,62]. On the other hand, the studies of Weaver and Abbas [23] and Atehnkeng et al. [24] showed a decline in the frequencies of biocontrol VCGs when follow-up treatments were delayed by one or two years. This suggests that the biocontrol carry-over effects may change from area to area, and the carry-over effects must continue to be investigated.

The current study revealed some unexpected results. In two areas (Figure 2), the prevalence of VCG IT006 in the untreated fields was comparable with the fields treated two years prior. The field-to-field variation in the microenvironment, agronomic practice, or predation by insects may have contributed to these observations [63].

The isolate of *A. flavus* MUCL 54911, belonging to VCG IT006, was identified and validated as the most efficient AF- strain among those included in the Italian fungal collection by Mauro and coworkers in 2013 and 2018 [28,59]. The study of Mauro et al. [28] highlighted the benefits of MUCL 54911, an active ingredient of AF-X1, in reducing the aflatoxin in maize. Mauro and colleagues showed that IT006 is the largest VCG in the Italian population, from which the active ingredient was chosen and was found in four out of five of the northern Italian regions where our current study was conducted. Areas 1 and 3 belong to the district of Rovigo, where 40% of the fields had been treated with AF-X1. The examined samples from Rovigo had larger proportions of IT006, suggesting that aerially dispersed and insect-transmitted conidia may be factors that facilitate the active ingredient movement [4,13]. The recovery of VCG IT006 in relatively high proportions in the untreated fields supports the approach of selecting VCGs native and well-adapted to the target regions for use as the active ingredients of biocontrol formulations for an improved persistence. Their adaptation to target areas plus their dispersal from treated to untreated fields are useful characteristics for biocontrol strains.

Data on cropping systems, such as rotation, soil texture, and other agricultural practices, might be relevant in explaining the observed variability among the fields. Several studies have examined the link between the previous crop and the *A. flavus* population [64,65,66]. In the prior studies, the highest densities of *A. flavus* were found in the soil after maize, followed by wheat, cotton, and sorghum. The results from the current study agree with these prior studies. One field treated two years prior to sampling with a prior crop of wheat had the lowest *A. flavus* density observed. Furthermore, soil texture is associated with the variability in *A. flavus* communities. Clay soil and *A. flavus* are positively correlated, while sandy soil is negatively correlated [65]. Even if not statistically significant, the lowest incidence of AF- isolates and those belonging to IT006 were detected in sandy soil. In area 1, in the current study, a field with sandy soil that had been treated with AF-X1 two years prior had a low incidence (35%) of IT006, with 84 CFU/g of the total *A. flavus* population (Figure 2a).

Conservation tillage combined with stalk burial, which increases the organic matter in the soil, were highly correlated with the *A. flavus* density and contributed to the maintaining of a reservoir of *A. flavus* [67,68]. In this study, significant differences were not detected, probably because so few (3 out of 28) fields had undergone conservation tillage, but the results from area 2 were in agreement with this statement; the density of *A. flavus* (251 CFU/g) was greater under no tillage with stalk burial than under tillage (109 CFU/g) in area 2 (Figure 1c).

In the present study, the sampling sites were chosen randomly to obtain diverse conditions. Therefore, a large variation in the cropping system may be a barrier to establishing a link between the cropping system and *A. flavus* density, as well as the occurrence of IT006. As expected, the carry-over experiment had no significant effect on the global fungal communities, other than on the proportions of the toxigenic and AF- *A. flavus* residing in the soil. Bhandari et al. [69] found that the application of the commercial biocontrol product FourSure™ had no overall impact on the microbiome composition of the treated and untreated crops. The aflatoxin biocontrol application has been reported to have no increase in *Aspergillus* density [24,39] and no influence on the composition of other mycotoxigenic fungal species such as *Fusarium*, and contamination with fumonisins [28,70].

The tracking of biocontrol active ingredients has been carried out by first classifying the *A. flavus* isolates by morphotype (L strain and S strain), and then conducting VCA in the L morphotype isolates with tester pairs specific to the VCGs of the active ingredients [16,26]. A qPCR technique has resulted in useful information on hazelnuts and pistachios because of its specificity, sensitivity, and accurate detection properties in accordance with the international EPPO standard (PM7/98) [54,57]. The usefulness of a qPCR in detecting AF- isolates based on lack of the *aflR* gene in the aflatoxin biosynthesis cluster was confirmed by the current study. This is the first study to track the abundance of biocontrol isolates in maize fields based on a qPCR of the mechanism underscoring one AF- genotype. However, non-aflatoxigenic strains exist with partial gene clusters that also lack *aflR*, so we cannot be certain that all the *aflR*-lacking strains detected during our qPCR were MUCL 54911. The current work found a predominance of AF- fungi in all the surveyed areas and at higher incidences in most fields in which a prior year biocontrol application was made. Similar results have been reported by Atehnkeng and coworkers [24] with other biocontrol fungi on small-holder farms in Africa. Our results show shifts in the *A. flavus* population following the application of the AF- *A. flavus* biocontrol product, AF-X1. Previously, this was demonstrated under various conditions, in both small-scale and large commercial-scale agriculture [23,24,26,71]. Nevertheless, a wide variability was observed among the studied fields. 

Examining the proportion of the biocontrol active ingredients post-application, over multiple years, is an important criterion to evaluate the success of *A.flavus* AF- strain-based biocontrol application. This study provides valuable data regarding the performance and stability of the active ingredient of AF-X1 in Italian agro-ecosystems for sustainable aflatoxin management. Additionally, this study confirms that post-application movement can occur to neighboring (untreated) fields and provide them with some level of protection from aflatoxin contamination. The proximity of neighboring fields, the area size, and the amount of the biocontrol applied are highly correlated with dispersal to untreated fields, as well as the persistence of the active ingredient over the time [24,72].

In conclusion, the use of aflatoxin biological control products with AF- *A. flavus* as their active ingredients is the most successful technique for aflatoxin management so far, demonstrating a considerable adaptability in the field with the strains native to the target regions. The current findings support the long-term durability of the application benefits. The primary detected influence of the AF-X1 applications is a switch in the *A. flavus* community structure towards increased incidences of AF- *A. flavus*. Based on our findings, as well as those from previous studies, the annual application of AF-X1 to commercial maize fields should be maintained, until more data are available that show the optimal timing and distribution of the applications that provide the most cost-effective treatments.

## 4. Material and Methods

### 4.1. Soil Sampling and Filed Data Collection

Soil was sampled in north Italy during April 2020 and 2021 in seven sampling areas, distributed across the provinces Rovigo, Modena, Padova, and Venezia. In each area, 4 fields (28 fields total) were chosen based on different time points involving AF-X1 application: once the previous year to sampling (treated n-1), once two years prior to sampling (treated n-2), both the previous year and two years prior to sampling (treated n-1 and n-2), or not at all (untreated). All the applications were made according to the label instructions of the farmers. For each treatment year, the crops were treated once at 25 kg/ha between the BBCH phenological growth stages 33–39 [73]. In each region, the approximate percentages of the maize farms where AF-X1 had been applied varied, with 40% in Rovigo, 35% in Padova, 30% in Venezia, and 25% in Modena.

In total, ten soil samples of ~50 g were collected with a surface-disinfected trowel, from the top 2 cm at 4 to 10 m intervals across diagonal transects of each of the 28 fields. The distances between the sampled fields exceeded 5 km [74,75]. The soil samples were taken to the laboratory, dried in forced air (40 to 45 °C, 48 h), and stored in plastic bags at 4 °C until processed. Additionally, information regarding the cropping system (e.g., the crop rotation, tillage system, stalk burial, and soil texture, provided by the farmers/extension agents) was collected for each the sampled fields. 

### 4.2. Aspergillus flavus Isolation

The isolation of *A. flavus* from the soil samples was performed aseptically, following the protocols previously reported [65]. Briefly, 10 g of soil per sample was mixed with 50 mL of double distilled sterile water and stirred for 20 min at 300 rpm A 100 µL aliquot of the soil suspension was transferred onto MRBA [76] and incubated at 31 °C for 3 d. The colonies of *A. flavus* were identified based on their morphology [77] and quantified as colony-forming units per g of soil (CFU/g). From each field, 10 to 15 discrete colonies of *A. flavus* were transferred to the low nutrient agar medium 5/2 (5% V-8 vegetable juice, 2% agar, pH 5.2) [78] and incubated (5–7 d, in the dark, 31 °C). The cultures were saved in sterile water vials at 4 °C containing five plugs (3 mm dia) of sporulating agar in 1 mL of sterile distilled water [78].

In total, 399 isolates (range = 10–15 per field) were used to quantify the persistence of the active ingredient of the biocontrol product AF-X1, MUCL 54911, using Vegetative Compatibility Analysis (VCA). The isolates were single spored (i.e., monosporic) through serial dilution on Malt Extract Agar (MEA) [59]. After 2 d of incubation at 31 °C, one colony per isolate was transferred to 5/2 agar. The single-spore transfers were performed in triplicate to ensure the culture purity. A total of five agar plugs from pure mature cultures were saved as above.

### 4.3. DNA Extraction

Monosporic *A. flavus* isolates (399 total) were used to evaluate the presence or absence of a section of the *aflR* gene, using a TaqMan qPCR assay developed for *A. flavus* [54]. In addition, all the isolates were subjected to a qPCR assay to evaluate the presence or absence of a section of the *aflR* gene. This gene is required for aflatoxin production. Positive (isolate FS7; aflatoxin producer) and negative controls (isolates FS3, FS5, FS6, and FV9; non-aflatoxin producers) were included. The 399 monosporic isolates were grown on Yeast Extract Sucrose Agar (YES agar) for 7 d at room temperature [79]. Fresh mycelium from the edges of the colonies were used to extract genomic DNA with the E.Z.N.A. fungal DNA mini kit (Omega Bio-Tek, Norcross, GA, USA), according to the manufacturer’s instructions. The DNA concentrations were measured with NanoDrop 2.0 (ThermoFisher, Wilmington, DE, USA) and adjusted to be less than 100 ng/µL [54].

### 4.4. qPCR Conditions

The two primers, AflF and AflR [54], were used at a concentration of 0.3 µmol, the TaqMan probe concentration was 0.1 µmol, with 1 × of TaqMan universal PCR MasterMix (Applied Biosystems, Loughborough, UK) and 1 µL of DNA (100 ng/µL) of the isolate being assayed. A StepOne thermal cycler instrument (Applied Biosystems, Loughborough, UK) was used to perform the reaction with the following cycle: an initial denaturation at 95 °C for 4.5 min, 40 cycles of 15 s at 95 °C, and 15 s at 60 °C. Each reaction was run in triplicate; positive and negative controls were included in each run. The standard curve utilized the DNA of *A. flavus* FS7 with serial dilution to test the qPCR sensitivity [54]. The AF- genotype of each isolate was assumed based on the CT value generated from the amplification curve of *aflR* gene and ranged from 20 to 47.61 (CT ≤ 35 = toxigenic; CT > 35 AF-).

### 4.5. Vegetative Compatibility Analysis (VCA)

To determine the distribution and frequencies of the AF-X1 active ingredient (MUCL 54911), all 399 monosporic isolates were subjected to VCA with the tester pairs of VCG IT006 [59], the VCG to which MUCL 54911 belongs, following the previously published protocols of Bayman and Cotty, [80] and Cotty [71].To obtain the nitrate non-utilizing (nit-) mutants, 10 µL of the spore suspension of each isolate was seeded into a well (3 mm diameter) in the center of SEL plates [71]. Sectors that were auxotrophic for nitrate were visible after 10 to 30 d of incubation at 31 °C. The auxotrophs were transferred to MIT, incubated for 3 d at 31 °C [51], transferred to 5/2 agar, and stored in water vials, as described above. Complementation tests with the tester pair of VCG IT006 were performed on starch medium [81]. In total, three wells (3 mm in diameter), 1 cm apart, were made in a triangular pattern in the center. A total of two wells were seeded with 10 µL of the spore suspension of each of the testers, and the third one was seeded with 10 µL of the spore suspension of the nit- mutant of the isolate being analyzed. The compatibility was assessed after 7 d of incubation at 31 °C. Wildtype growth at the zone of mycelial interaction indicated that the isolate belonged to VCG IT006 [80]. 

### 4.6. Data Analysis

Data on the CFU/g of the total fungi and *A. flavus* in the soil samples were ln transformed and data on the percentage of the AF- isolates and those belonging to IT006, both computed on all the 399 recovered *A. flavus* isolates and on the aflatoxin-free *A. flavus*, were arcsin transformed before a statistical analysis was performed to reduce the heterogeneity in the variance. All data obtained were subjected to a univariate analysis of variance (ANOVA) using the generalized linear model (GLM) procedure, and significant differences between the means were determined using Tukey’s HSD test (α = 0.05). The statistical package IBM SPSS statistics 27 (IBM Corp., Armonk, NY, USA) was used for the data analysis.

## Figures and Tables

**Table 1:** List of maize fields sampled in this study. Location (district and municipality), sampling year, geographic coordinates (latitude and longitude), AF-X1 treatment regimen (untreated, treated n-2, treated n-2&n-1, and treated n-1; n is the sampling year), soil texture, crop rotation (one or two years before sampling), and stalk burial were reported.

Sampling Year	Area N ^1^	District ^2^	Municipality ^3^	Latitude	Longitude	Treatment ^4^	Soil Type	Rotation 1 ^5^	Rotation 2 ^6^	Tillage	Stalk Burial
2020	1	Rovigo	Occhiobello	44.9297388	11.62801768	Untreated	Silt	Maize	Tomato	Till	No
2020	1	Rovigo	Occhiobello	44.943139	11.567212	n-2	Sand	Maize	Maize	Till	No
2020	1	Rovigo	Occhiobello	44.951586	11.547809	n-1&n-2	Silt	Maize	Maize	Till	No
2020	1	Rovigo	Occhiobello	44.955789	11.554532	n-1	Silt	Maize	Tomato	Till	No
2020	2	Rovigo	Occhiobello	44.969438	11.70443	Untreated	Silt	Wheat	Pea	No till	No
2020	2	Rovigo	Occhiobello	44.93645466	11.62733917	n-2	Clay	Wheat	Maize	No till	No
2020	2	Rovigo	Fiesso Umbertino	44.955438	11.589537	n-1&n-2	Silt	Maize	Maize	No till	Yes
2020	2	Rovigo	Occhiobello	44.94879764	11.60269669	n-1	Silt	Maize	Wheat	Till	Yes
2020	3	Rovigo	Occhiobello	44.94469842	11.49092054	Untreated	Clay	Wheat	Wheat	Till	No
2020	3	Rovigo	Occhiobello	44.94834217	11.59027712	n-2	Sand	Wheat	Maize	Till	Yes
2020	3	Rovigo	Fiesso Umbertino	44.973695	11.630536	n-1&n-2	Silt	Maize	Maize	Till	Yes
2020	3	Rovigo	Occhiobello	44.96235125	11.6690985	n-1	Silt	Maize	Soybean	Till	Yes
2021	4	Modena	Finale Emilia	44.81483	11.217337	Untreated	Clay	Wheat	Maize	Till	No
2021	4	Modena	Finale Emilia	44.829477	11.094888	n-2	Silt	Wheat	Maize	Till	No
2021	4	Modena	Finale Emilia	44.835933	11.27449	n-1&n-2	Sand	Maize	Maize	Till	No
2021	4	Modena	Finale Emilia	44.86689	11.174496	n-1	Silt	Maize	Wheat	Till	No
2021	5	Rovigo	Occhiobello	44.98778	11.698756	Untreated	Clay	Soybean	Wheat	Till	No
2021	5	Rovigo	Occhiobello	44.931735	11.629024	n-2	Silt	Soybean	Maize	Till	No
2021	5	Rovigo	Occhiobello	44.958125	11.627436	n-1&n-2	Silt	Maize	Maize	Till	Yes
2021	5	Rovigo	Occhiobello	44.971765	11.721178	n-1	Silt	Maize	Wheat	Till	Yes
2021	6	Padova	Noale	45.54925	12.052739	Untreated	Silt	Soybean	Soybean	Till	Yes
2021	6	Padova	Noale	45.549233	12.05297	n-2	Silt	Soybean	Maize	Till	Yes
2021	6	Padova	Noale	45.553166	12.045033	n-1&n-2	Clay	Maize	Maize	Till	Yes
2021	6	Padova	Noale	45.552759	12.048152	n-1	Clay	Maize	Soybean	Till	Yes
2021	7	Venezia	Scorzè	45.563136	12.10695	Untreated	Silt	Soybean	Soybean	Till	Yes
2021	7	Venezia	Scorzè	45.569842	12.099649	n-2	Silt	Soybean	Maize	Till	Yes
2021	7	Venezia	Scorzè	45.565978	12.09769	n-1&n-2	Silt	Maize	Maize	Till	No
2021	7	Venezia	Scorzè	45.56353	12.10323	n-1	Silt	Maize	Soybean	Till	No

^1^ Number of areas, 3 areas in 2020 and 4 areas in 2021. ^2^ 4 provinces in which sampled fields were located. ^3^ Different areas where the soil samples were collected from the surveyed fields. ^4^ Prior AF-X1 treatment and n refer to the sampling year. ^5,6^ Crop rotation for one and two years prior to sampling, respectively.

**Table 2:** Results of ANOVA run for colony-forming units (CFU/g) of total fungal community and of *A. flavus* resident in the soil samples collected from 3 areas in 2020 and 4 areas in 2021. The 7 different areas belong to 4 districts: Modena, Rovigo, Padova, and Venezia. Per each area, 4 different AF-X1 treatment regimens were considered: untreated, treated n-2, treated n-2&n-1, and treated n-1; n is the sampling year. Percentage of AF- strains on all *A. flavus* isolated was also reported, so was the percentage of the VCG IT006 on total *A. flavus* strains and on the AF- strains.

Sample Year	Treatment Year	N. of Isolates	Replicates ^1^	Total Fungi (CFU/g)	*A. flavus* (CFU/g)	% AF- ^2^	IT006 on *A. flavus* % ^3^	IT006 on *A. flavus* Atox % ^3^
2020				*	*	NS	NS	NS
	Untreated	39	3	702 b	51 c	77	37	48
	2018	44	3	704 b	163 ab	75	41	56
	2018 + 2019	42	3	778 b	190 a	93	72	77
	2019	44	3	2688 a	86 b	86	77	88
2021				NS	NS	NS	**	**
	Untreated	60	4	1229	96	38	12 b	23 b
	2019	54	4	964	63	75	62 a	86 a
	2019 + 2020	60	4	863	84	68	57 a	79 a
	2020	56	4	943	138	72	54 a	74 ab
2020, 2021 Combined				NS	*	*	**	**
	Untreated	99	7	1003	77 b	55 b	23 b	34 b
	Treated 1 yr	198	14	1271	111 a	77 ab	58 a	76 a
	Treated 2 yrs	102	7	826	130 a	79 a	63 a	78 a

^1^ Each replicate is a separate commercial field. ^2^ The % of AF- was calculated based on the total number of isolates of *A. flavus p* (120 and 279 isolates of *A. flavus* recovered in 2020 and 2021, respectively). ** (*p* < 0.01), * (*p* < 0.05); NS= not significant; different letters indicate significant difference according to Tukey’s HSD test. ^3^ Percentages were calculated based on the total number of isolates collected for each treatment.

## Data Availability

Not applicable.
